# Supplementary material for: Untethered muscle tracking using magnetomicrometry
Source: Front Bioeng Biotechnol. 2022 Oct 25;10:1010275. doi: 10.3389/fbioe.2022.1010275 (PMC9640962; doi:10.3389/fbioe.2022.1010275)
Supplement: Supplementary file 2 [file DataSheet1.pdf]

frontiers in Bioengineering and Biotechnology | Biomechanics

Original Research Article

Supplementary Material

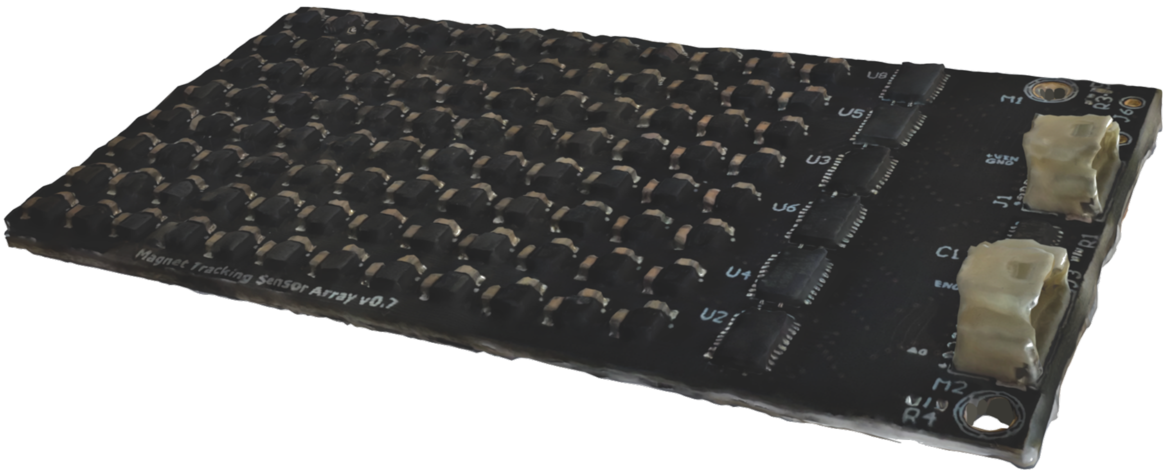

Supplementary Figure 1: 3D Scan of the Magnetomicrometry Sensor Board. *See supplementary folder 3_D_Scan_of_MM_Sensor_Board for a 3-D scan of the magnetomicrometry sensor board in .obj format with associated reference files (.mtl, .png).*

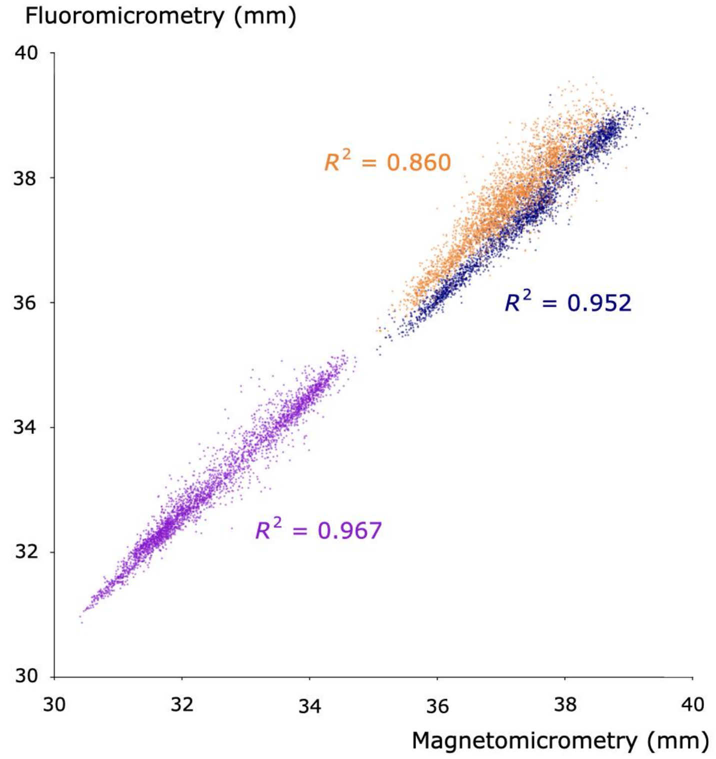

Supplementary Figure 2: Coefficients of Determination (R^2 values) between Magnetomicrometry and Fluoromicrometry. *We compared all magnetomicrometry measurements (horizontal axis) across 50 gait cycles of turkey running (10 gait cycles at each of 5 speeds, for each bird) against time-synchronized, interpolated fluoromicrometry measurements (vertical axis). Data are plotted in blue, orange, and purple for Birds A, B, and C, respectively. Coefficients of determination (R^2 values, shown in corresponding colors) for each bird were 0.952, 0.860, and 0.967, respectively.*

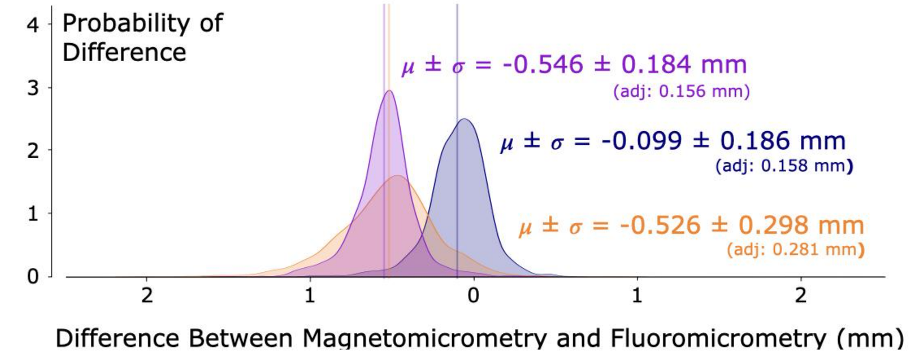

Supplementary Figure 3: Kernel Density Estimates of Differences between Magnetomicrometry and Fluoromicrometry. *We subtracted time-synchronized, interpolated fluoromicrometry measurements from magnetomicrometry measurements across all 50 gait cycles of turkey running (10 gait cycles at each of 5 speeds, for each bird). Kernel density estimates show the distribution (vertical axis) of these differences (horizontal axis), with data plotted in blue, orange, and purple for Birds A, B, and C, respectively. Vertical lines indicate mean offsets (in corresponding colors), with the mean offsets and standard deviations labeled. Adjusted standard deviations that compensate for fluoromicrometry noise (0.098 mm, standard deviation) indicate an estimate of the magnetomicrometry measurement noise.*

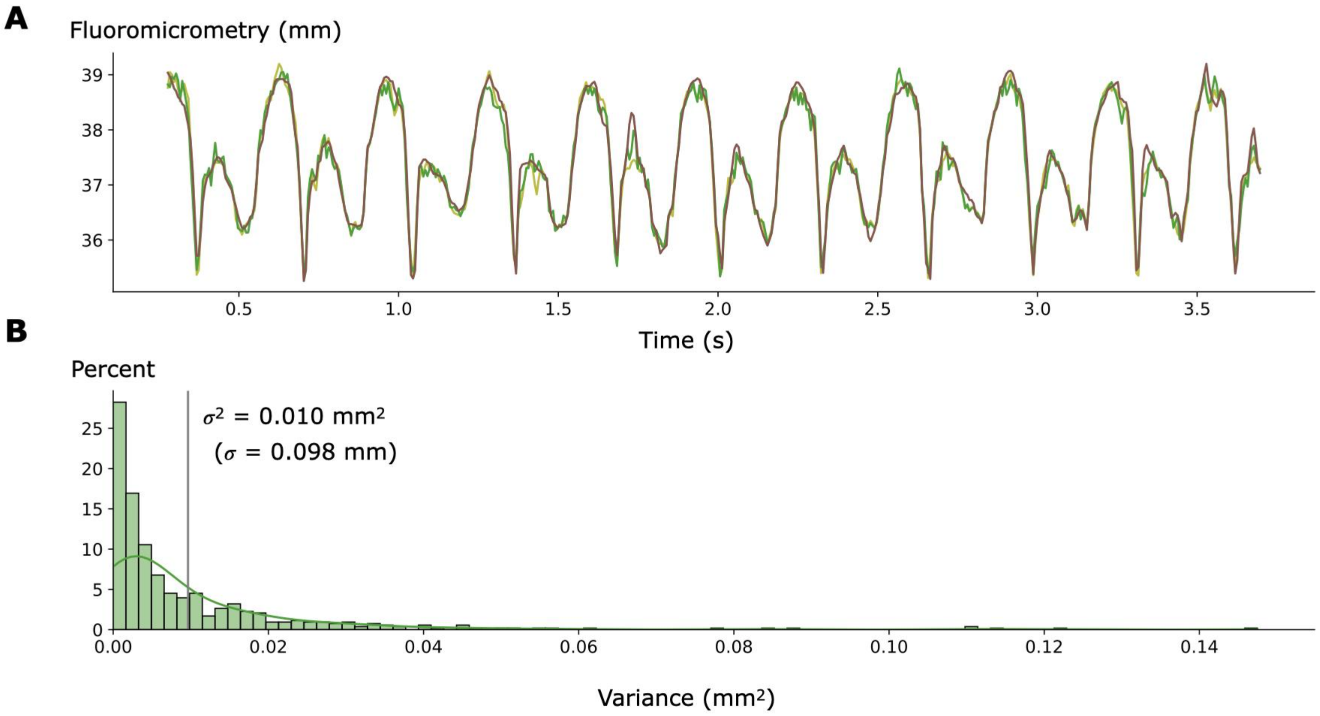

Supplementary Figure 4: Study-Specific Limitations to the Use of Fluoromicrometry. (A) *Ten gait cycles of raw fluoromicrometry video data (Bird A, 3.5 m/s), independently manually re-labeled three times, are shown in light green, dark green, and brown.* ***(B)*** *The histogram shows the distribution (vertical axis) of the variance (horizontal axis) between the three manual processed fluoromicrometry signals at each timepoint of the data, with a kernel density estimation curve overlay. A vertical line indicates the average variance, 0.010 mm^2, and the corresponding square root of the variance is 0.098 mm.*

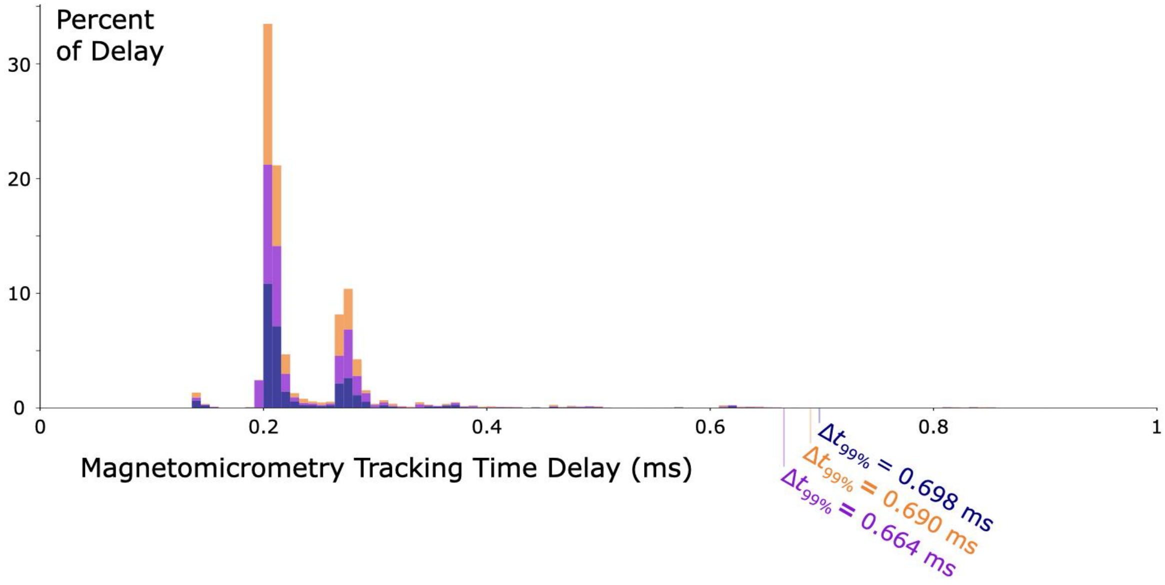

Supplementary Figure 5: Magnetomicrometry Tracking Time Delay. *The magnet tracking computer recorded the times it received the magnetic field data and the times it completed the magnet tracking algorithm. The distribution (vertical axis) of the difference between these times (horizontal axis) is the tracking time delay and indicates the bandwidth at which magnetomicrometry can track the muscle tissue length. The data are shown as a stacked histogram, with blue, orange, and purple data corresponding to Birds A, B, and C, respectively. Data are from all turkey gait cycles used to compare magnetomicrometry against fluoromicrometry. The 99th percentile time delay ($\Delta t_{99\%}$) is labeled for each bird. The ninety-ninth percentile time delay for all birds was less than one millisecond.*

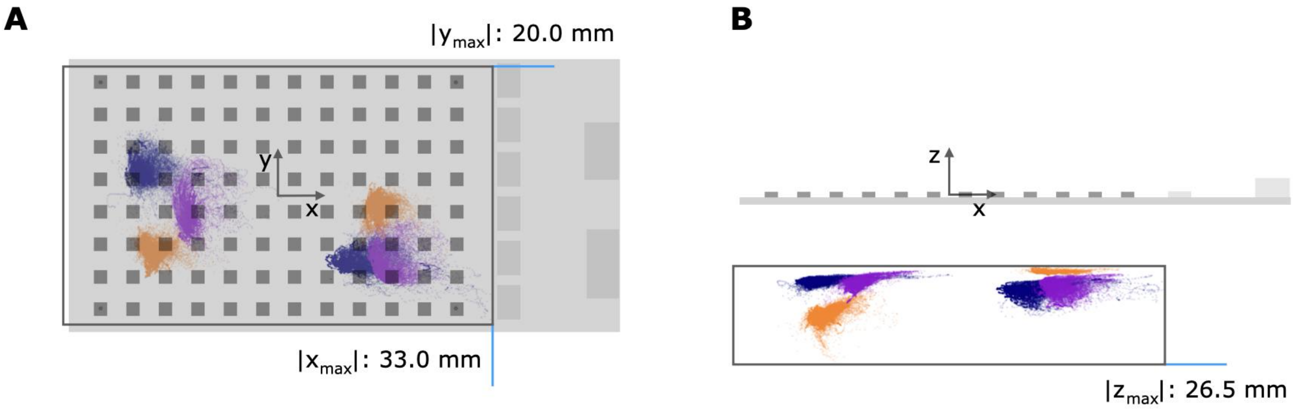

Supplementary Figure 6: Extent of Bead Positions During Ramp Ascent and Descent and Vertical Ascent and Descent. *Analysis of the magnetic bead position data (shown) recorded during all variable terrain activities revealed the extent of the magnetic bead positions during the tracking. We used these bead position maximums to design a benchtop test (see supplementary Figure 7) to verify the validity of our magnetomicrometry measurements during the variable terrain activities.*

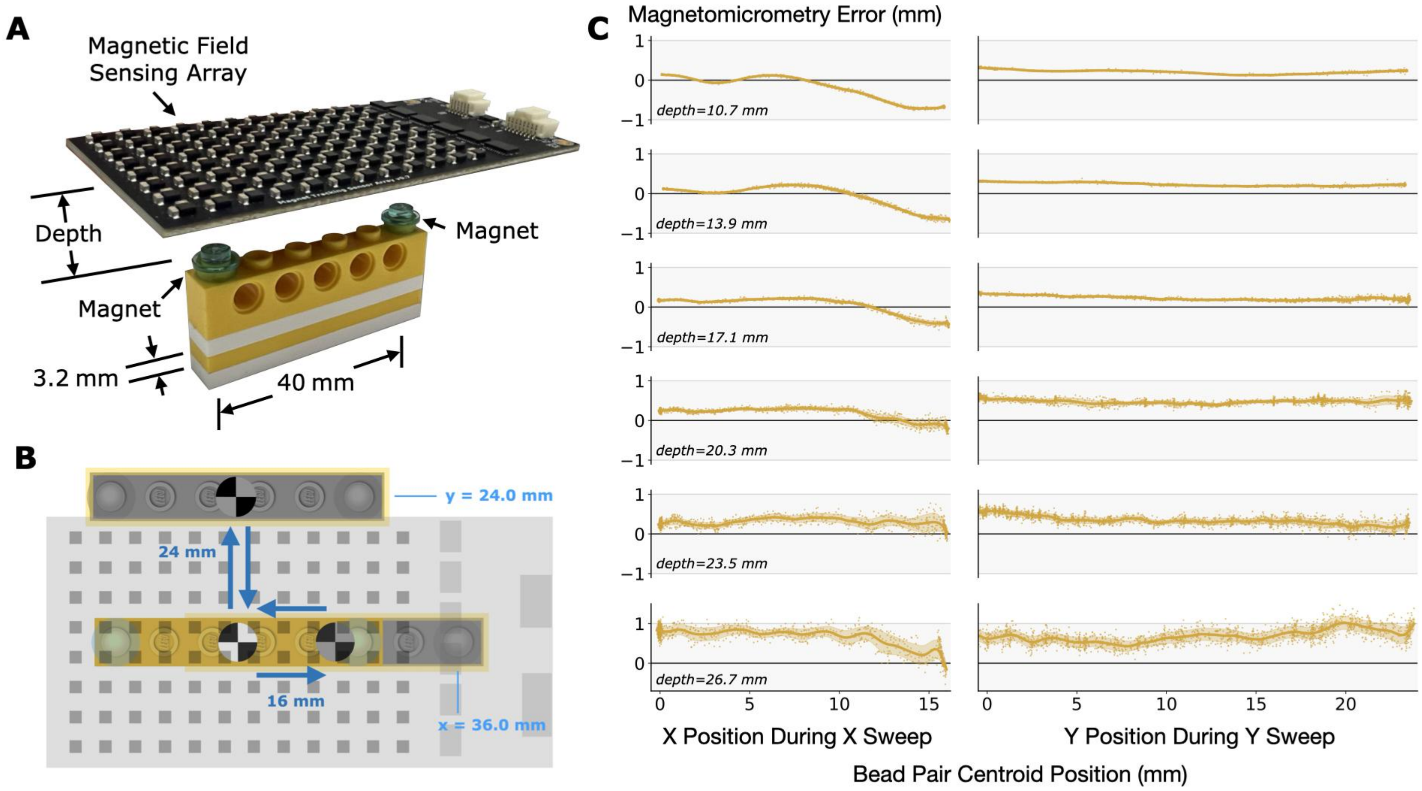

Supplementary Figure 7: Benchtop Magnetomicrometry Validation. (A) *Two magnets were placed 40 mm apart in a 1x6 LEGO Technic block and centered under the sensing array. We used the tracked magnet z-position as a guide in setting up the minimum depth measurements, and we enforced the remaining depths by adding/removing 3.2-mm-thick 1x6 plates under the Technic block.* ***(B)*** *We manually swept the magnets out and back to center along the x and z axes. We set the sweep trajectory to sweep just beyond the volume within which the magnets were tracked during the variable terrain activities (see Supplementary Figure 6). For reference, the centroid of the two beads is labeled at the origin and at the extent of the sweep trajectories.* ***(C)*** *The vertical axis represents the magnetomicrometry error, and the horizontal axes represent the centroid x and y position for the two sweep trajectories at each depth. The submillimeter error range is marked with a gray background. A maximum error of 1.463 mm was found at a test location just beyond the tracked bead position extent (bottom right plot).*

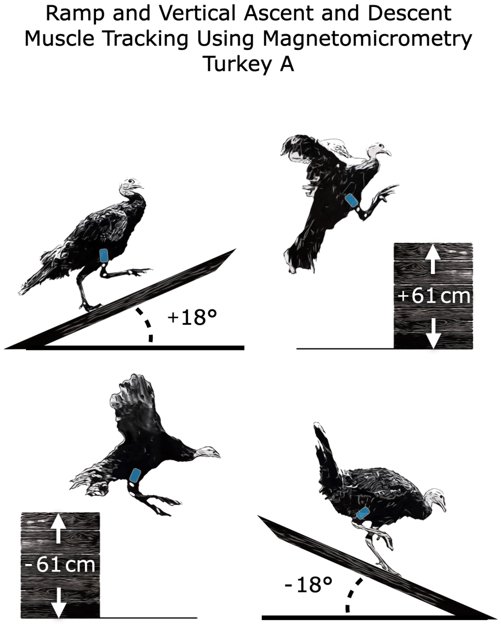

Supplementary Movie 1: *Turkey A. Completed ramp ascent, vertical ascent, vertical descent, and ramp descent with the real-time magnetomicrometry signal overlaid.*
